# Evaluation of Human Settlement Environment and Identification of Development Barriers Based on the Ecological Niche Theory: A Case Study of Northern Shaanxi, China

**DOI:** 10.3390/ijerph20031772

**Published:** 2023-01-18

**Authors:** Zhicheng Zhang, Bin Fang, Xiuqing Li, Yirong Wang

**Affiliations:** 1 School of Geography Science, Nanjing Normal University, Nanjing 210023, China; 2 Research Center of New Urbanization and Land Problem, Nanjing Normal University, Nanjing 210023, China; 3 Jiangsu Provincial Geographic Information Resources Development and Utilization Cooperative Innovation Center, Nanjing 210023, China; 4 School of Business, Xi’an University of Finance and Economics, Xi’an 710100, China; 5 School of Science and Engineering, Xi’an Siyuan University, Xi’an 710038, China

**Keywords:** ecological niches, human settlement environment, loess plateau, obstacle

## Abstract

The quality of human settlement environment (HSE) is related to people’s well-being. Since the implementation of the Western Development Strategy and the Grain to Green Program, the HSE in northern Shaanxi has undergone a major transformation. In order to explore the evolution pattern and seek a coordinated development strategy for all systems in the whole region, this paper, from the perspective of “production–living–ecological”, evaluates the HSE niche breadth of northern Shaanxi based on the ecological niche theory, analyzes its spatial differentiation characteristics, and identifies the development barrier factors, with the help of ArcGIS spatial analysis tools and the barrier degree model. It is found that: from 2000 to 2020, (1) the niche breadth of HSE in northern Shaanxi is high in the north and low in the south, showing obvious spatial unevenness; (2) the development of transportation promotes the improvement of HSE, but also intensifies the spatial unevenness, and the uncoordinated development rate of transportation and production and living systems has seriously restricted the further development of HSE; (3) the niche breadth of the ecosystem for each county is much lower than that of the production and living systems, and the ecological environment becomes the short board of the improvement of HSE in northern Shaanxi. Based on the patterns and problems found in the study, this paper proposes a strategy to improve the HSE of northern Shaanxi by prioritizing the balanced development of production, living systems, and transportation, strictly implementing the concept of ecological priority, dynamically adjusting the hierarchy of policies, vigorously optimizing the industrial layout, and focusing on the joint improvement of the human settlement environment in the whole region. This study expands the theories and evaluation methods of HSE to a certain extent, and the results have guiding values for promoting the sustainable development of HSE in northern Shaanxi and even the whole Loess Plateau region.

## 1. Introduction

Human settlement environment (HSE) is the place where human beings live together, the surface space of the earth closely related to human survival activities, the base on which human beings live in nature, and the main place where human beings use and transform nature [1]. Faced with the global problems of urban overcrowding, environmental degradation, and housing shortage that have been prevalent since the Industrial Revolution, as well as chronic poverty and backwardness in the rural areas of developing countries, the Greek scholar Doxiadis proposed “Ekistics” in the 1950s [2]. This concept is considered to be the origin of human settlement science, and it emphasizes the view of human settlements as a complete system [3]. In 1947, Kaseva first outlined findings on sustainable human settlement development and management based on research into waste management [4], which informed subsequent related research. The establishment of the United Nations Center for Human Settlements (UNCHS) and the UN Commission on Human Settlements in 1978 led to the widespread development of HSE. In 2002, the establishment of the United Nations Human Settlements Programme (UN-HABITAT) marked a further focus on HSE as a global development issue. As the research continued, the key words of HSE began to trend toward social problem orientation, policy orientation, and practice [5]. The impact of living conditions on health [6], the evaluation of HSE quality in urban fringe areas in the context of urban–rural integration [7], and the spatial evolution characteristics of urbanization and urban systems in different regions [8] are the key concerns of human settlement science. In recent years, with the continuous improvement of HSE, people have higher requirements for realistic living conditions, and some issues such as ecological disorders are widely viewed by the international community as a cause for concern. The theme of the UN World Habitat Day 2021 is “Accelerating Urban Action”, and China has incorporated carbon peaking and carbon neutrality into the overall layout of ecological civilization, hoping to create a resilient, inclusive, safe, and diverse urban environment to ensure a safe living space for everyone [9].

In order to achieve the common goal of HSE research, scholars often take the evaluation of livability as the entry point of the research. The object of the evaluation of HSE livability is divided into subjective object [10], which is the perceived subject of the environment, i.e., the residents [11,12], and objective object [10], which is the description of various factors influencing the livability of the HSE [13,14]. In terms of evaluation system, scholars have established several evaluation index systems based on different evaluation needs, scales, and objects. The World Health Organization (WHO) summarized livable conditions as safety, health, convenience, and amenity [15]. The Chinese scholar Wu [1] divided the human settlement into five subsystems: natural, human, social, residential, and support. There are also many studies whose evaluation index systems are constructed based on natural–human systems. For example, Guan et al. constructed a two-dimensional system of natural and human indicators to evaluate the comprehensive suitability of urban habitat in Liaoning Province [16]. With the introduction of the “production–living–ecological” (PLE) theory, scholars have tried to evaluate HSE from this perspective, including livability, resilience, and vulnerability [17]. For example, Tang et al. constructed a HSE evaluation index system for typical tourist cities based on an in-depth analysis of the connotation of the PLE system, and explored the HSE evolution process and mechanism using Zhangjiajie City as an example [18]; Chen et al. constructed a PLE evaluation index system to study the spatio-temporal evolution pattern and driving forces of urban vulnerability using the Harbin-Changchun urban agglomeration as an example [19]; Yang et al. analyzed the evolution and optimization path of rural settlement characteristics in Shanghai based on the PLE function [20]. The PLE system is a specific field formed by the interaction between human society and natural ecology, which is inseparable from HSE [21,22,23,24]. Livability is the expression of comfortable living, production is the prerequisite for livability, and ecology is the well-being of livability. The proposal and development of PLE provides a new perspective for HSE research.

In terms of methods for evaluating HSE, linear weighting and hierarchical synthesis are the current mainstream methods [25]. For example, based on HSE theory and resilience theory, Wang et al. [26] constructed a HSE resilience evaluation index system and evaluated the rural HSE resilience in the western part of Chongqing; Feng et al. [27] constructed a HSE evaluation index system from both natural and human dimensions, and evaluated the comprehensive livability of HSE in Inner Mongolia Autonomous Region. In recent years, in order to have a deeper understanding of the status and role of each component unit in natural and social systems as well as the dynamics of system evolution and development, the application of ecological niche and the niche trend theory has gradually gone beyond ecology [28] and penetrated into the fields of urban geography and urban economy, becoming an effective tool for human research on social systems since the late 20th century [29], which has provided important guidance for research on competition mechanisms, adaptation of ecological elements (including humans) to the environment, human ecology, and urban ecology [30]. The ecological niche theory can also be introduced into HSE research. The niche of HSE can be defined as the sum of natural factors (e.g., climate, hydrology, geology, geomorphology) and social factors (e.g., labor conditions, living conditions, ecological relationships) that are provided to humans by the natural–social system within a certain geographical area [31]. It can also be defined as the HSE measurement of the diversity, quantity, and efficiency of social, economic, and natural resources utilization within a certain geographical range. It can reflect the adaptability of the current HSE to various human economic and productive activities, as well as the nature, function, status, and role of the HSE component units and their demographic and resource strengths and weaknesses. The ecological niche theory can be a good measure of the “freeze frame” of continuously changing ecological relationships [28]. The introduction of ecological niche is a supplement to the existing HSE theory, and the relevant methods also have important implications for HSE evaluation. Based on the ecological niche theory, it is possible to quantitatively analyze the competitive ability of HSE in terms of the PLE of each geographical unit and scientifically predict the development trend of regional HSE, which provides a new perspective to promote the realization of the habitat goal of developed productivity, convenient living, and good ecology.

Although scholars have produced rich results in the field of HSE, most of the existing studies have focused on developed areas [32,33,34] or on suburban or rural–urban areas that are pivotal in the urbanization process [26], and relatively little attention has been paid to less developed areas. With rapid economic development and the effects of national strategies and policies, the HSE in less developed regions has also undergone significant improvements. Northern Shaanxi is one of these regions. Since the implementation of the Grain to Green Program (GGP) in 1999, the area of woodland and grassland in northern Shaanxi has increased significantly, the ecological environment has continued to improve [35], and the value of all types of ecosystem services has increased [36]. At the same time, with the overall strengthening of agricultural infrastructure, the district’s comprehensive agricultural production capacity has also been further improved. The industry in this area has grown rapidly based on resource-based industries, absorbing a large number of people in employment, and has now formed an economic characteristic dominated by resource-based industries. Under multiple strategic measures such as the Western Development Strategy (WDS), GGP, and Precise Poverty Alleviation (PPA), the quality of HSE in northern Shaanxi has undergone a major transformation. However, increasingly frequent environmental modification activities have also deepened the impact on natural systems, leading to serious challenges to the sustainability of HSE [37]. For example, while road hardening and land expansion for construction provide convenience for humans, they also destroy the original natural landscape, leading to ecological degradation and a reduction in the value of ecosystem services [38].

In summary, in order to explore the HSE evolution pattern in less developed regions and seek strategies for the coordinated development of each system, this paper takes northern Shaanxi as an example, based on HSE theory, PLE theory, and ecological niche theory, and uses the niche breadth model, hot spot analysis, spatial auto-correlation, and the barrier degree model to assess the niche of HSE, analyze its spatial differentiation characteristics and the trade-off relationship within the PLE system, and identify the barrier factors for HSE enhancement in the hope of proposing targeted HSE optimization strategies. The introduction of ecological niche theory and the perspective of production–living–ecological are the main innovations of this paper, which expands the relevant theories and evaluation methods of HSE to a certain extent. The results provide some guidance in achieving sustainable development of HSE in a typical region such as northern Shaanxi.

## 2. Materials and Methods

### 2.1. Study Area

**Figure 1:**
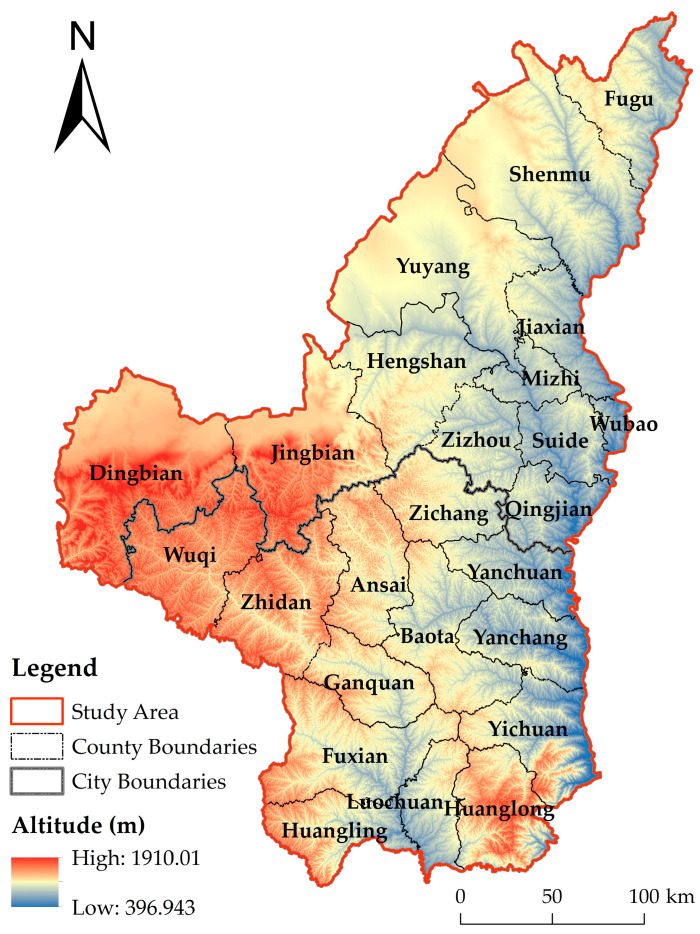
Schematic diagram of the study area.

Northern Shaanxi is located between Shanxi, Inner Mongolia, Gansu, Ningxia, and the Guanzhong Basin, and is the central part of China’s Loess Plateau, which is also the key planning area of the Western Development Strategy and the Belt and Road Strategy, with a geographical area of 35°02′–39°25′ N, 107°15′–111°15′ E. It has a total area of about 8.03 × 10^4^ km^2^ and includes 2 prefecture-level cities, Yulin and Yan’an, with 21 counties and 4 municipal districts under its jurisdiction (Figure 1).

The topography of the region is low in the south-east and high in the north-west, and the landscape types include Loess Plateau and Wind-Sand Transition Zone, with little rainfall and drought. Severe disturbance caused by human activities has made northern Shaanxi one of the most serious areas of soil erosion in China and even in the world. Since the implementation of the GGP in 1999, northern Shaanxi has become the region with the largest continuous greening in China, and the rate of soil erosion has been reduced [39]. In terms of productivity, the crude business model and the fragmentation of arable land have led to a low profitability of agricultural production and a serious loss of farming population [40]; however, being an important energy base in China (coal reserves of 600–100 million tons, oil reserves of more than 600 million tons, and natural gas reserves of more than 30,000 billion cubic meters) [41], industry in northern Shaanxi has been able to develop rapidly, thus allowing it to gradually develop from a typical agricultural and pastoral area into a transitional area between agriculture and mining [42], and its GDP per capita is even higher than the national average. Northern Shaanxi is also one of the transportation hubs in the Loess Plateau region, with the Baoxi Railway, Baomao Expressway, Qingyin Expressway, and several provincial roads passing through the region, but the complex geomorphological features create serious obstacles to the further development of transportation levels. In addition, a low level of productivity and urbanization, a small size of the central city, and a large difference in income between urban and rural areas also exist in the region to varying degrees.

The typical topography, special geographical location, backward agriculture, and a single type of industry make the development of HSE in northern Shaanxi quite different from the developed eastern coastal areas of China. Evaluating the quality of the region’s HSE and identifying constraints in its development process can help plan solutions tailored to local conditions and can also provide lessons for other similar regions.

### 2.2. Data Sources

Considering that the Grain to Green Program and the Western Development Strategy, implemented in 1999 and 2000, respectively, might have a greater impact on the HES in northern Shaanxi, and taking into account the availability of data, this paper takes 2000 as the starting year and 2020 as the cut-off year of the study. At the same time, to facilitate comparative research, this paper selects 2010 as the time point, which divides the research period into two decades. The data are obtained as follows. (1) Indicator data are calculated from the statistical data of *China County Statistical Yearbook*, *Yulin City Statistical Yearbook*, and *Yan’an City Statistical Yearbook* in 2000, 2010, and 2020, as well as the statistical bulletin and water resources bulletin of each district and county. In the case of data inconsistency, national statistical data shall prevail. (2) Land use data are taken from the Resource and Environmental Science and Data Center of the Chinese Academy of Sciences (https://www.resdc.cn (accessed on 5 October 2022)). (3) DEM were downloaded from 91 Guardian software, with an accuracy of 30 m × 30 m. (4) PM 2.5 data (0.01° × 0.01°) were obtained from Washington University in St. Louis (https://sites.wustl.edu/acag/datasets/surface-pm2-5/#V5.GL.02 (accessed on 8 August 2022)).

### 2.3. Evaluation Methods of Human Settlement Environment

#### 2.3.1. Construction of Evaluation Index System

Based on the analysis of the connotation of HSE and PLE, summarizing the previous achievements and combining with the situation of the study area, this paper constructs a three-dimensional evaluation index system of production–living–ecological for evaluating HSE ecological niche. The production environment is the foundation of socio-economic development and the key to the enhancement of human well-being. It is concerned with food security as well as the efficiency, intensity, and trends of development, and can be characterized by the scale, efficiency, level, concentration of development, and trends of agricultural production and non-agricultural production [43]. The living environment relies on considerable income and convenient public services. The income ratio of urban and rural residents is used to measure the level of urban–rural oneness; the total retail sales of social consumer goods per capita is used to characterize the consumption capacity of residents; and the density of transportation land, the number of hospital beds per 10,000 people, and the elementary school teacher–student ratio are used to characterize the service capacity of three major public facilities: transportation, medical care, and education. Ecological environment is an important condition to support and satisfy human production and development, and it is a fundamental element to maintain the health of human habitat [44]. Ecosystem services can express the ability of ecosystems to provide “products” or “services” to human society; precipitation and habitat abundance index can reflect the positive results of afforestation in northern Shaanxi in the past few decades; PM 2.5 is a common source of pollution in northern China, mainly caused by human economic activities [27], and it is used as a negative indicator to reflect the damage caused by human activities to the natural environment.

In summary, a total of 16 indicators were selected in this paper to evaluate the HSE ecological niche in northern Shaanxi (Table 1). The results of the co-collinearity test show that the VIF values of these indicators are less than 10, indicating that the model results are less affected by co-collinearity [45]. Meanwhile, due to the complexity and uncertainty of the HSE system, gray correlation analysis is therefore used to quantitatively measure the correlation between indicators. This method is less demanding on data and can reduce the loss caused by system asymmetry. The results show that the gray correlation coefficients of all indicators are above 0.80, and the change trends among the indicators are basically the same, indicating that the indicators selected in this paper could act on the HSE ecological niche in cooperation with each other. In addition, given that HSE is a complex giant system, in order to overcome the subjectivity of assigning weights, this study adopts the entropy value method to calculate the weights to reflect the intrinsic structural relationship of the index system. The entropy value method, which determines objective weights mainly based on the magnitude of variability of indicators, has been widely used in several fields. Generally, the smaller the information entropy of an indicator is, the greater the variability of the indicator value is, and the more information the indicator can provide, i.e., the greater the weight of the indicator. Conversely, a higher information entropy of an indicator means a lower weight. The detailed steps of the entropy value method can be found in [26].

#### 2.3.2. Niche Breadth Model

According to ecological niche theory, the niche breadth indicates the degree of resource utilization of a species [47]. For this study, the HSE niche breadth indicates the resource utilization efficiency and the degree of resource dominance of each county in HSE development. A high value of niche breadth in a county indicates that the county is highly efficient in resource utilization, has obvious resource advantages, and is competitive in HSE enhancement; a low value indicates that the county is weak in competitiveness.

Drawing on the niche trend model [48], this paper uses the “state” of production, living, and ecological resources available to each county, i.e., the quality of HSE, to represent the current niche breadth, and the “trend” of resources available to each county, i.e., the rate of HSE quality change, to represent the future niche breadth. The quality of HSE is obtained by weighting the indicators in Table 1. The combination of “state” and “trend” can reflect the niche breadth of HSE in each county and its contribution to the quality improvement of HSE in northern Shaanxi. The formula is as follows:

(1) $$N_{i}=\frac{S_{i}+A_{i}P_{i}}{\sum_{j=i}^{n}\left(S_{j}+A_{j}P_{j}\right)}$$

where *i*, *j* = 1, 2, 3, …, *n*; *N_i_* is the niche breadth of county *i*; *S_i_* and *P_i_* are the state and trend of county *i*, respectively; *S_j_* and *P_j_* are the state and trend of county *j*, respectively; *A_i_* and *A_j_* are the dimensional conversion coefficients; *S_j_* + *A_j_P_j_* is called the absolute niche.

#### 2.3.3. Hot Spot Analysis

The Getis–Ord Gi* index of Hot Spot Analysis in Spatial Statistics Tools in ArcGIS was used to perform the cold and hot spot local correlation analysis of the niche breadth of HSE. The formula is as follows:

(2) $$G_{i}^{*}=\frac{\sum_{j}^{n}W_{ij}\left(d\right)x_{i}}{\sum_{j}^{n}x_{j}}$$

(3) $$Z\left(G_{i}^{*}\right)=\frac{G_{i}^{*}-E\left(G_{i}^{*}\right)}{\sqrt{Var\left(G_{i}^{*}\right)}}$$

where *W_ij_* denotes the spatial weight; *E*($G_{i}^{*}$) and *Var*($G_{i}^{*}$) are the mathematical expectation and standard deviation of $G_{i}^{*}$, respectively. If *Z*($G_{i}^{*}$) is positive and significant, it belongs to the high value spatial agglomeration, i.e., the hot spot area of the niche breadth; conversely, if *Z*($G_{i}^{*}$) is negative and significant, it belongs to the low value spatial agglomeration, i.e., the cold spot area of the niche breadth.

In addition, since the hot spot analysis requires a minimum sample size of 30, the niche breadth of the counties calculated in this paper was gridded with a grid size of 5000 m × 5000 m and a number of 3437.

### 2.4. Analysis Method of Spatial Trade-Offs within PLE System of HSE

Exploratory Spatial Data Analysis (ESDA) mainly detects the dependence of the spatial distribution pattern of things and phenomena, determines the diffusion, polarization, or randomness of the spatial distribution, and is used to reveal the mechanism of interaction between the property values of the research object [49]. The bivariate local spatial autocorrelation analysis model proposed by Anselin [50] improved the defects of the previous spatial autocorrelation analysis with only one variable. Accordingly, by choosing the niche breadth of one function as the independent variable and the spatial lag of another function’s niche breadth as the dependent variable, the trade-off relationship and degree between the two functions could be analyzed.

### 2.5. Barrier Degree Model for HSE Enhancement

The barrier degree model is a mathematical statistical method to effectively determine the key factors that hinder the development of things, and it is now widely used in the study of impact factors in various disciplines. With reference to the existing research [26], the barrier degree model of HSE enhancement is constructed based on the evaluation index system of the HSE niche in northern Shaanxi. The formula is as follows:

(4) $$P_{j}=\left[\left(1-Z_{j}\right)\times W_{j}/\overset{m}{\underset{j=1}{\sum }}\left(1-Z_{j}\right)\times W_{j}\right]\times 100\%$$

(5) $$V_{j}=\sum P_{j}$$

where *P_j_* is the barrier degree of the single indicator to HSE enhancement; *Z_j_* is the standardized value of the single indicator; *m* = 16; *V_j_* is the barrier degree of the criterion layer to HSE enhancement.

## 3. Results

### 3.1. Spatial Differentiation of HSE Niche

**Figure 2:**
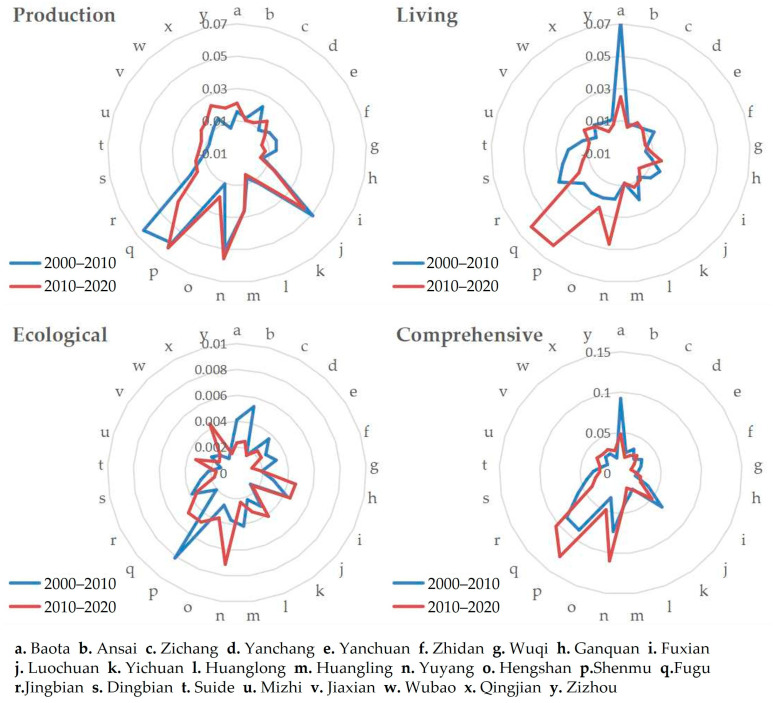
Production–living–ecological niche breadth change of human settlement environment in northern Shaanxi from 2000 to 2020.

**Figure 3:**
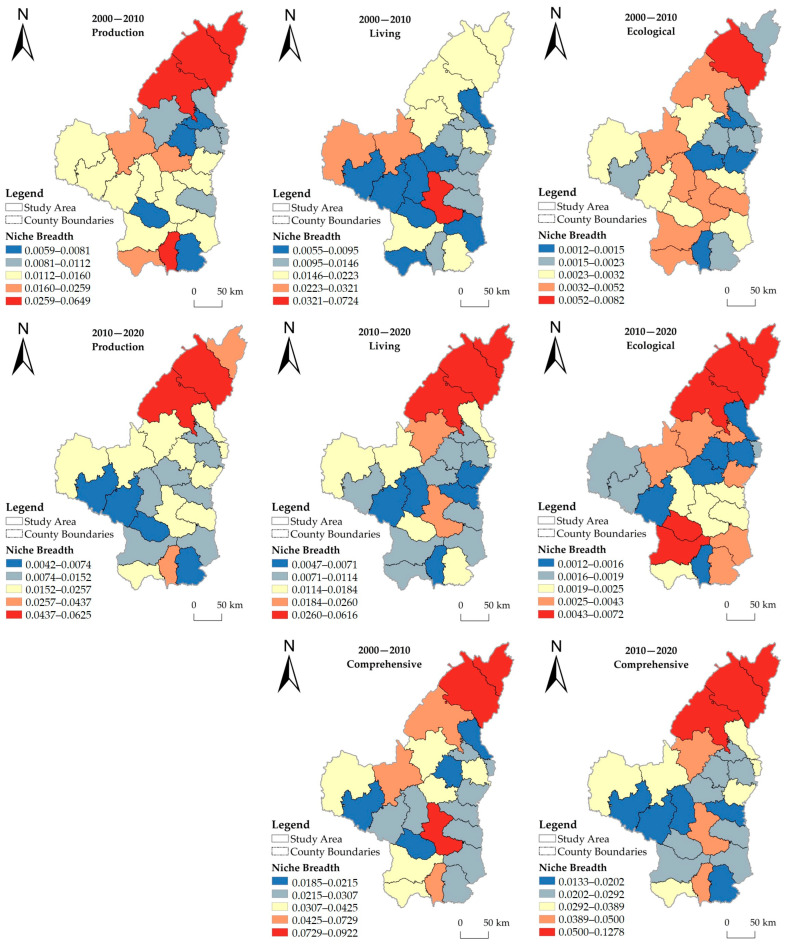
Spatial distribution of niche breadth of human settlement environment in northern Shaanxi from 2000 to 2020.

The spatial differentiation pattern, temporal evolution process, and regional characteristics of the production–living–ecological niche breadth of HSE in 25 counties in northern Shaanxi from 2000 to 2020 are obvious (Figure 2 and Figure 3). The details are as follows.

Production environment: During the study period, the production niche breadth of each county is basically stable, showing the spatial distribution characteristics of high in the north and low in the south. Specifically, from 2000 to 2010, Fugue, Shenmu, Luochuan, and Yuyang had much higher niche breadth values than the other counties, while all other counties had similar values. From 2010 to 2020, although the value of Fugu decreased significantly, from the highest to the fourth highest, Fugu, Shenmu, Luochuan, and Yuyang still had values above all other counties. Over the 20 years, the production niche breadth increased slightly in all the counties of Yulin City, except for Fugu County. However, in Yan’an City, only Baota District and Yanchuan County saw a slight increase, while the rest of the counties saw a decrease in production niche breadth to varying degrees.

Living environment: Unlike the production environment, the living niche breadth in northern Shaanxi varied more markedly in space during the study period. From 2000 to 2010, the living niche breadth in the study area shows a circle-like spatial distribution. Baota District is the center of the circle, and the value of its living niche breadth is much higher than that of other counties; in the middle of the circle are the counties immediately adjacent to Baota District, and their values are at the lowest level of the study area; the counties in the north and south ends of the study area are at the outermost part of the circle, and the values of these counties are in the middle for the study area. From 2010 to 2020, the values of living niche breadth in northern Shaanxi change significantly in spatial distribution, which can be summarized as high in the north and low in the south. Specifically, the value of Baota District slightly decreased, and the values of its surrounding counties remained at the lowest level in the study area; the high value of living niche breadth shifted from Baota District to Yuyang, Shenmu, and Fugu, which are located in the northernmost part of the study area.

Ecological environment: The ecological niche breadth in northern Shaanxi was much lower than that of production and living environment during 2000–2020, and the change in its value is also small, but the change of spatial characteristics is obvious. From 2000 to 2010, the highest values were found in Shenmu, followed by Ansai; the low-value areas were mainly concentrated in the eastern part of the study area; Luochuan and Huanglong, located in the south, and Wuqi, located in the west, also had low values; the rest of the counties were at medium or high levels. From 2010 to 2020, Yuyang, Shenmu, and Fugu, located in the northern region, and Fuxian and Ganquan, located in the southern region, had the highest values; the values in the western part of the study area decreased significantly, and the eastern part remained as a low-value concentration area.

The comprehensive evaluation results show that from 2000 to 2020, the niche breadth of HSE in northern Shaanxi has a small temporal variation, but the spatial variation in the north–south direction is obvious, and the overall distribution is high in the north and low in the south. In the north of the study area, Yuyang, Shenmu, and Fugu have the highest values, and in the south, Baota has the highest value, while the peripheral counties in Baota have the lowest level of niche breadth in the study area.

**Figure 4:**
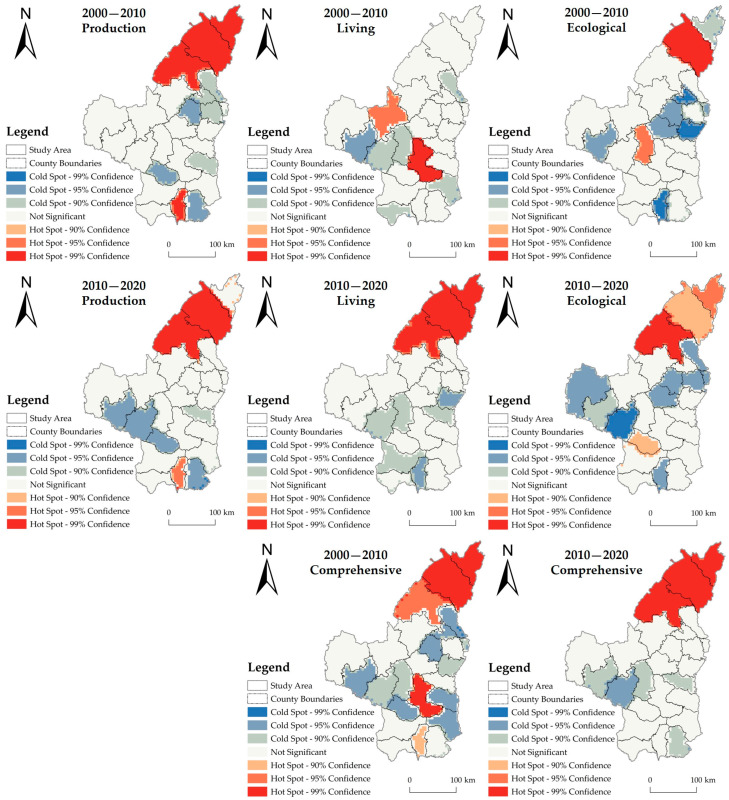
Hot spot distribution of human settlement environment niche.

The hot spot analysis (Figure 4) helps to understand more clearly the local correlation characteristics of the HSE niche in northern Shaanxi. The results show that from 2000 to 2020, the hot spots of the production niche clustered in the northern part of the study area, and the cold spot areas shifted from the eastern to the southwestern part of the study area. Living niche hot spots change significantly, with hot spots mainly clustering in Baota District from 2000 to 2010, but from 2010 to 2020, hot spots clustered in the three northern counties (Shenmu, Fugu, and Yuyang). From 2000 to 2020, the cold spots of the living niche clustered in the periphery of Baota District. The hot spots of the ecological niche from 2000 to 2020 clustered in the northern part of the study area, while the cold spot clustering areas shifted from the east to the west. Overall, the hot spots of HSE in northern Shaanxi were mainly concentrated in the three northern counties during the study period, and the cold spot agglomerations were mainly distributed around Baota District.

### 3.2. Spatial Trade-Offs within PLE System of HSE

To deeply explore the spatial trade-offs among production, living, and ecological, the bivariate local autocorrelations of niche breadth within the PLE system from 2000–2010 and 2010–2020 were analyzed with the help of GeoDa 1.20, using a 5000 m grid as the base unit and *Z*-test (*p* = 0.05) as the criterion. With the help of the LISA distribution map, the spatial interaction of two adjacent functions in the region can be better expressed [51].

**Figure 5:**
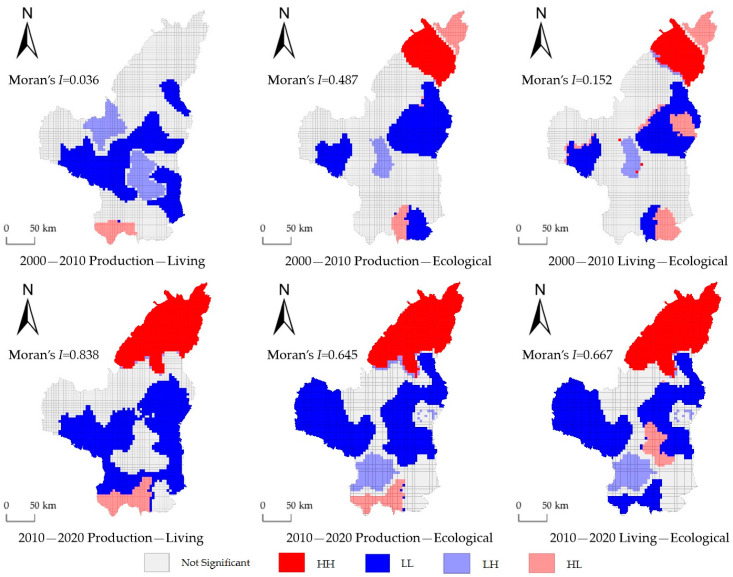
Bivariate local LISA expressions for PLE systems.

The trade-off relationship within the PLE system is spatiotemporally heterogeneous (Figure 5). Specifically, (1) from 2000 to 2010, the main synergy type of the P-L system was LL, which was concentrated in the periphery of Baota District, and the LH trade-off was concentrated in Baota District. From 2010 to 2020, the spatial trade-offs of the P-L system changed significantly, with HH synergy occurring in the three northern counties (Yuyang, Shenmu, and Fugu), while LL synergy was still concentrated in the periphery of Baota District but with a wider distribution than before. It is noteworthy that the P-L system in Huangling County was in the HL trade-off from 2000 to 2020. (2) The changes in the P-E system mainly manifested in the expansion of the synergistic zone. From 2000 to 2010, HH synergy was present only in Shenmu, while Fugue County, also in the northern part of the study area, presented HL trade-offs; LL synergy was mainly distributed in the eastern counties, Wuqi County in the west, and Huanglong County in the south. From 2010 to 2020, the synergistic area expanded spatially, i.e., all three counties in the northern part of the study area showed HH synergy, while the LL synergistic areas in the east and west kept extending to the central part of the study area and were nearly connected. Throughout the study period, the P-E system in Luochuan County in the southern part of the study area was in HL trade-off. (3) The evolution of spatial trade-offs in the L-E system is similar to that of P-E, mainly showing the spatial expansion of synergistic areas. From 2000 to 2010, HH synergy appeared only in Shenmu, and Fugu presented HL trade-off; LL synergy was mainly distributed in the eastern counties of the study area, Wuqi County in the west, and Luochuan County in the south. From 2010 to 2020, HH synergy expanded to the three counties in the north, LL synergy expanded from the east and west to the middle, Luochuan and Huangling in the south of the study area also showed LL synergy, HL synergy clustered in Baota District, and LH synergy clustered in Fuxian County.

Overall, from 2000 to 2020, the HH synergistic area of P-L system, P-E system, and L-E system expanded in the three northern counties of the study area or shifted to this range; LL synergy extended from the east and west sides of the study area to the central part; HL trade-off, LH trade-off, and LL synergistic were interspersed in the southern region.

### 3.3. Barriers to HSE Enhancement

#### 3.3.1. Barrier Factor Analysis

The barrier degree of each factor to HSE enhancement in northern Shaanxi was calculated with the help of the barrier degree model, and the top three factors for each county were listed (Table 2).

Statistics found that (1) from 2000 to 2010, the main barrier factors for HSE enhancement in 19 counties were transportation land density (TLD), total ecosystem services value (TESV), and the number of industrial enterprises above the scale (NIEAS). Except for Baota District, the factor with the highest barrier degree in each unit was TLD. In addition to the three factors mentioned above, the ratio of social security spending to GDP (RSS) had a large impact on the enhancement in Baota, Huangling, Yuyang, Shenmu, Fugu, and Jingbian. (2) From 2010 to 2020, TLD was still the most dominant barrier factor in most counties. The main barrier factors in Baota District changed significantly, from TESV, NIEAS, and RSS to TLD, TESV, and RSS. The three northern counties also changed significantly, with the primary factor changing to RSS, and grain yield per unit area (GYPU) and annual precipitation (AP) becoming important barrier factors.

Overall, HSE enhancement in most counties in northern Shaanxi continued to be constrained by transportation and ecosystem services, and only a few counties saw changes in the major barrier factors over the 20-year period. In comparison with the results of the niche breadth measurement and the PLE spatial trade-off analysis, significant changes were still observed in Shenmu, Fugu, and Yuyang in the northern part of the study area, and in Baota in the southern part.

#### 3.3.2. Barrier Degree Analysis of PLE

**Figure 6:**
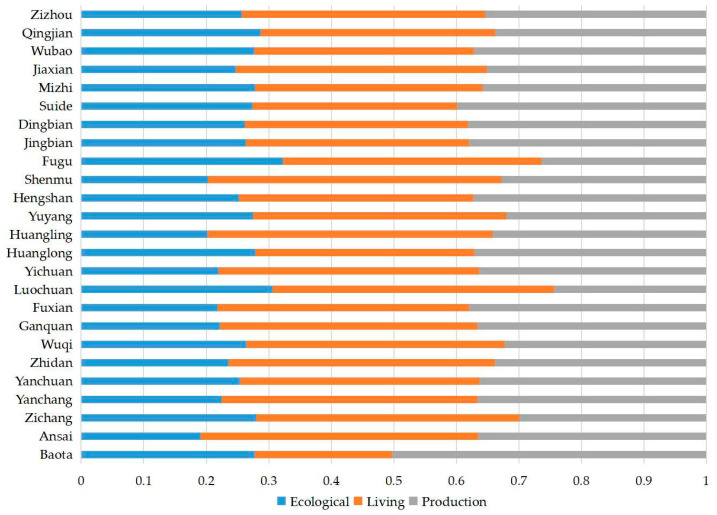
Barrier degrees of production–living–ecological on the enhancement of human settlement environment in northern Shaanxi from 2000 to 2010.

**Figure 7:**
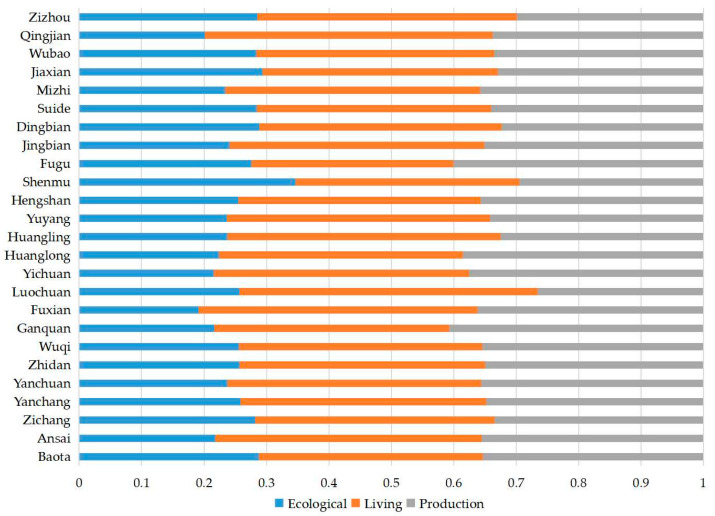
Barrier degrees of production–living–ecological on the enhancement of human settlement environment in northern Shaanxi from 2010 to 2020.

Based on the results of the barrier degree measurement, the barrier degree map of the PLE system of northern Shaanxi HSE enhancement was drawn (Figure 6 and Figure 7).

From 2000 to 2010, the HSE niche of more than half of the counties in northern Shaanxi was mainly constrained by the development level of living environment, and the barrier degrees produced for Shenmu, Huangling, and Luochuan were all greater than 0.45. The main barriers for Baota and Suide were production environment, with barrier degrees of about 0.50 and 0.40, respectively. Nine counties, including Yanchuan, Fuxian, Huanglong, and Hengshan, were constrained by both production and living environments, and the barrier degrees of both systems were above 0.35. The ecological environment had stronger constraints on Fugu, Luochuan, Zichang, and Baota.

From 2000 to 2020, the barrier degree of the PLE system to HSE in northern Shaanxi changed to some extent, but living environment constraint or L-P double constraint was the main pattern. The production environment produced serious constraints only in a few areas. Ecological, P-E, and P-L-E constraint patterns did not appear.

## 4. Discussion

### 4.1. Formation of Spatial Differentiation of HSE Niche

With the help of the niche breadth model and hot spot analysis, we found that the spatial distribution of HSE niche breadth in northern Shaanxi is characterized by being high in the north and low in the south. The values of Yuyang, Shenmu, and Fugu are the highest in the northern region, and the values of Baota are the highest in the southern region. The low-value areas are clustered in the periphery of Baota District. The reasons for the formation of this feature are complex and varied, and we will discuss it from the perspective of the PLE environment.

Agricultural production and industrial economy are direct manifestations of the production environment, and it may be possible to find some reasons for the formation of spatial differentiation of HSE in terms of their changes, i.e., in terms of changes in the production environment itself. In the industrial economy, as economic powerhouses in western China, the energy and chemical industries in Shenmu and Fugu have been ranked among the top in the country in terms of production capacity and output efficiency. The economic benefits from industrial production have laid a solid foundation for HSE enhancement in the northern part of the study area. With regard to agricultural production, taking Yulin City as an example, relevant studies [52] show that the arable land area in the northern part of Yulin City did not change significantly from 2000 to 2018, which provides food security for the region to a certain extent, while the southern counties saw a decrease in arable land area due to ecological engineering measures such as the GGP carried out in the Loess Hills and Ravines. Some external factors, such as the tilt of national strategies and differences in resource endowments among counties, could also lead to spatial unevenness in the niche breadth of the production environment. For example, during the 13th Five-Year Plan period, Yan’an City increased investment in coal-rich areas such as Fuxian County, Huangling County, and Baota District [53], which made the industrial development of other counties in the city relatively backward. It can be seen that the spatial unevenness of the production system in northern Shaanxi, such as differences in natural resources, policies, capital investment, industrial base, and national strategies, has led to significant differences in the resource utilization capacity and efficiency of the counties in the process of HSE enhancement, which has further resulted in the spatial distribution feature of high niche in the north and low niche in the south.

The living environment is also closely related to the spatial characteristics of HSE in northern Shaanxi. Transportation is an important grasp of economic development [54], and likewise a major factor in the development and change of the living environment of HSE. Since the implementation of China’s Western Development Strategy (2000), the level of transportation in northern Shaanxi has undergone a qualitative change, with the dynamic development level of highways in all of northern Shaanxi and Yan’an City exceeding that of the Guanzhong region around 2010, and Yulin City exceeding the average level of Shaanxi Province [55]. The dynamic development level of highways in northern Shaanxi has become absolutely superior. A study [56] showed that the contribution of transport development to the economy in northern Shaanxi gradually increased since 2000, especially in Yulin, where the contribution of transport to economic development and social transformation increased most rapidly. This might be one of the reasons for the shift of the high-value area of the living environment niche breadth to the northern part of the study area from 2000 to 2020. Equalization of public service facilities is an important element to achieve spatial environment optimization and social equity [57] and a necessary condition to create a living circle that is good for living, working, traveling, and learning [58]. The formation of the spatial distribution characteristics of HSE niche in northern Shaanxi is also closely related to it. For example, Shenmu has made great efforts to develop health care services and has even implemented a universal free medical care policy [59]. With the reduced burden of health care, the income growth of residents has been boosted to some extent, which in turn has a positive impact on the living niches of Shenmu.

Since the implementation of the GGP, the ecological environment in northern Shaanxi has been significantly improved [60], and the service capacity of the ecosystem has been increased, which has a positive effect on HSE that cannot be ignored. It is noteworthy that the ecological niche breadth in the three northern counties located in the Wind-Sand Zone along the Great Wall was higher than that in other areas, and especially the changes in Fugu County were most obvious during the study period. This is basically consistent with the results of the study by Ye et al. [36]. Based on the niche theory and the basic principles of the niche trend model, it can be inferred that the reason for this result might be that the ecological background of the three northern counties is much more fragile than that of other regions, and as the key implementation area of the program, its ecological environment has improved much more than that of other regions.

Production, living, and ecological environment are not separate. There are intertwined situations between them [61]. For example, Qingjian County in Yulin City seized the opportunity of the Precise Poverty Alleviation strategy and used industry as a channel to help nearly 30,000 people increase their income by 2018, with an annual per capita income increase of 3522 yuan [62], narrowing the urban–rural income gap and improving the quality of life of residents to a certain extent. In this process, the production environment is enhanced (industrial development) first, and the living environment is subsequently benefited (urban–rural income gap is reduced). For this study, the spatial unevenness of economic development is the core factor in the formation of the spatially heterogeneous characteristics of the HSE niche breadth in northern Shaanxi, while economic development mainly benefits from industrial development and national strategies and policies, i.e., the production environment dominates the spatial characteristics of the HSE niche in the study area. On this basis, the living environment such as transportation, infrastructure, and public service facilities can be improved, and the improved living environment has positive feedback on the production environment. Although the niche breadth of the ecological environment slightly increased during the study period, its value was at a lower level compared with production and living, and it played an extremely limited role in HSE enhancement. Because of this, the ecological environment has become the short board of HSE enhancement in northern Shaanxi.

### 4.2. Constraint and Enhancement

From the perspective of barrier factors, transportation, ecosystem services, number of large-scale industrial enterprises, and social security expenditures were the main barrier factors for HSE enhancement in most counties in northern Shaanxi from 2000 to 2020. The positive response of economic development to the improvement of transportation level directly drives the enhancement of the HSE’s production and living environment. Since the Western Development Strategy, the rapid development of transportation in northern Shaanxi has contributed to the formation of the HSE niche spatial pattern. However, the contribution of transportation is slightly insufficient compared with the dominant indicators such as industrial output, food production, and economic density. The difference in the development rate of transportation and other factors might be the reason for this result. The amount of ecosystem services, as another important barrier factor, constrains the HSE niche enhancement in various ways. On the one hand, the fragility of the ecological background in northern Shaanxi was the main shortcoming of HSE enhancement. Although it improved over the 20 years, the service capacity and service volume of the improved ecosystem remained at a relatively low level. On the other hand, in order to adapt to the rapid economic development, ecological land has been continuously squeezed by urban and rural construction land, and some of the ecological benefits brought by the Grain to Green Program have been offset. A related study [50] showed that from 2000 to 2005, the area of ecological land such as woodland and grassland in northern Shaanxi grew, but after 2005, the growth momentum of ecological land slowed down and gradually moved toward shrinkage. However, the ecological environment is the environmental condition necessary to ensure the operation of production and life, and it is able to regulate the direction of development of the production and living environment [42,63]. Any sustainable production and living system needs the support of ecosystems, and if the latter deteriorates, it will directly constrain the former [41]. Therefore, the extremely limited growth of ecosystem service capacity and the fragility of the native ecological background in northern Shaanxi not only restrict the enhancement of the HSE ecological niche, but also further hinder the production and living environment. The measurement results show that two indicators, the number of large-scale industrial enterprises and the ratio of social security spending to GDP, also constrain the improvement of the HSE niche. Since embarking on industrial transformation, the Chinese government and managers at all levels in northern Shaanxi have emphasized the need to implement concepts such as efficient and regulated production as well as ecological priorities and have restructured some enterprises and even shut down unregulated and polluting industrial enterprises on a large scale. For example, in 2009, the Shaanxi provincial government issued the second coal mine closure list for Yulin City, with 58 coal mines being required to shut down. These measures have led to a sharp reduction in the number of large-scale industrial enterprises, but improved ecological environment, intensive use of resources, and increased production efficiency have instead allowed northern Shaanxi’s economy to continue to grow rapidly, and HSE has been enhanced as a result. The constraints of the ratio of social security expenditure to GDP on HSE are also mainly reflected in the “value”. To avoid wasting social resources, social security expenditures have been kept within an elastic range for a long time, while the economy of northern Shaanxi has grown rapidly, which has led to a decreasing ratio. Therefore, the decrease in the number of large-scale industrial enterprises and the decline in the ratio of social security spending to GDP should not be counted as major constraints.

In terms of the barrier degree of P-L-E environment, there are mainly two patterns of single living environment barrier and living-production dual barrier for the enhancement of HSE in northern Shaanxi. Combining the barrier degrees at the indicator level, it can be concluded that the constraints arising from the production environment are caused by the decrease in the number of large-scale industrial enterprises, and the constraints of the living environment are mainly caused by the ratio of social security expenditures to GDP and transportation factors. Influenced by transportation factors, the constraint of living environment on HSE enhancement in northern Shaanxi becomes more prominent after excluding the number of large-scale industrial enterprises and the ratio of social security expenditure to GDP. In addition, it is worth noting that the measurement results show that the ecological environment has not produced significant constraints. This is due, on the one hand, to the trade-off between the ecological environment and the living and production environment, and on the other hand, to the fact that the niche breadth of the ecological environment has been at an extremely low level, even producing differences in order of magnitude compared with the production and living environment. Because of this, for northern Shaanxi, the ecological environment is an important shortcoming for HSE enhancement. From the above discussion, it is easy to find that the results of the barrier model are generally in line with the reality of the study area, but the limitations of the method lead to the barrier of some factors being only reflected in the “value”. In other words, with socio-economic development, some of the factors are no longer significant barriers to HSE improvement, but they still seem to have some influence from the model’s arithmetic results. Therefore, future studies should not only carefully consider whether the selected indicators or impact factors can be adapted to socio-economic development, but also closely integrate the situation of the study area when analyzing the results to remove unreasonable factors. This will provide a more scientific basis for the proposed strategy.

In summary, differences in economic base, resource endowment, and policies have caused spatial unevenness in the PLE niche breadth for HSE in northern Shaanxi. Different development rates of transportation and economy have further exacerbated this unevenness, and even made the transportation level an important barrier factor for HSE enhancement. The fragile native ecological environment and the dramatic expansion of urban and rural construction land have led to the contribution of the Grain to Green Program to HSE becoming limited. In addition, the trade-offs within the PLE system have made it challenging to improve HSE overall. Due to the dynamic and complex nature of the HSE system, when carrying out enhancement optimization, we should distinguish the main and secondary issues, prioritize the main issues while taking into account the shortcomings, and dynamically coordinate multiple secondary issues in the optimization process. For northern Shaanxi, the mismatch in development speed between transportation and production and living environment is the main factor that restricts HSE enhancement, which should be adjusted as a priority. The fragility of the ecological environment has been an inherent shortcoming of the region, and the concept of ecological priority should be consistently implemented. For example, land should be used as intensively as possible when enhancing the production and living environment in order to avoid large encroachments on ecological land and to maximize the ecological benefits brought by various measures. In addition, the imbalance should be broken by optimizing policies and industrial layout and other means to drive the development of low-value areas for HSE niche breadth, thus realizing HSE enhancement in the whole area of northern Shaanxi.

## 5. Conclusions

Based on niche theory and PLE theory, this paper evaluated the HSE niche of each county in northern Shaanxi from a macro perspective with the help of the niche breadth model, ArcGIS hot spot analysis tool, and barrier degree model, analyzed its spatial distribution characteristics and the barrier factors for enhancement, and put forward development suggestions from the perspective of breaking through the constraints. The main conclusions are as follows.

1. From 2000 to 2020, the overall spatial characteristics of HSE niche breadth in northern Shaanxi showed a pattern of high in the north and low in the south, and the niche breadth value of ecological environment was much lower than that of production and living environment. The rapid development of the energy industry, which has brought about substantial economic growth, was the main reason for the increase in the HSE niche. The differences in resource endowment and economic base, as well as the strategic layout of the government, etc., have led to an uneven level of industrial development among the regions, resulting in the spatial unevenness of the HSE niche in northern Shaanxi, i.e., the production environment has dominated the formation of the spatial characteristics of the HSE niche in northern Shaanxi.
2. The hot spots of HSE niche breadth in northern Shaanxi mainly concentrated in Shenmu, Fugu, and Yuyang, which are located in the northern part of the study area, and the cold spots mainly concentrated in the periphery of Baota District. From 2000 to 2020, the HH synergies of P-L, P-E, and L-E also mainly clustered in the three northern counties, while the LL synergies showed an overall extension from the eastern and western parts of the study area to the central part. In the southern part, the spatial trade-offs of the PLE system were intricate and complex, with HL trade-offs, LH trade-offs, and LL synergies interspersed.
3. The development of transportation has driven the enhancement of HSE in northern Shaanxi, but it has also exacerbated the spatially uneven characteristics of the niche. At the same time, the difference in the development rate of transportation and production and living environment is an important reason to restrict the enhancement of HSE niche in northern Shaanxi, and the ecological environment is an inherent shortcoming of the enhancement.

Finally, based on the results of this paper and the actual situation in northern Shaanxi, we propose a strategy to improve HSE by prioritizing the balanced development of production, living systems, and transportation, strictly implementing the concept of ecological priority, dynamically adjusting the hierarchy of policies, vigorously optimizing the industrial layout, and focusing on the joint improvement of the human settlement environment in the whole region.

## Figures and Tables

**Table 1:** Evaluation index system of the niche of human settlement environment.

Target Layer	Criterion Layer	Index Layer	Property	Weight
Niche of human settlements in northern Shaanxi	Production environment	Economic density	+	0.1031
		Grain yield per unit area	+	0.1060
		Land reclamation rate	+	0.0515
		Average industrial output per land	+	0.1088
		Number of industrial enterprises above the scale	+	0.1138
		Contribution rate of tax revenue	+	0.0172
	Living environment	Urban–rural income ratio	−	0.0099
		Total retail sales of consumer goods per capita	+	0.0718
		Ratio of social security spending to GDP	+	0.0744
		Transportation land density	+	0.2040
		Number of hospital beds per 10,000 people	+	0.0388
		Elementary school teacher–student ratio	+	0.0263
	Ecological environment	Total ecosystem services value	+	0.0285
		Annual precipitation	+	0.0132
		Habitat abundance index	+	0.0188
		PM 2.5	−	0.0139

Note: “+” indicates that the indicator plays a positive role, “−” indicates that the indicator plays a negative role. Habitat abundance index = Ai Bo × (0.35 × forest land + 0.21 × grassland + 0.28 × wetland + 0.11 × cropland + 0.04 × construction land + 0.01 × unused land)/total regional land area [26]. The total ecosystem services value was measured using the equivalent value method [46].

**Table 2:** The main barrier factors for enhancement of human settlement environment in northern Shaanxi.

	2000–2010			2010–2020		
	Factor 1	Factor 2	Factor 3	Factor 1	Factor 2	Factor 3
Baota	TESV	NIEAS	RSS	TLD	TESV	RSS
Ansai	TLD	TESV	NIEAS	TLD	TESV	NIEAS
Zichang	TLD	TESV	NIEAS	TLD	TESV	NIEAS
Yanchang	TLD	TESV	NIEAS	TLD	TESV	NIEAS
Yanchuan	TLD	TESV	NIEAS	TLD	TESV	NIEAS
Zhidan	TLD	TESV	NIEAS	TLD	TESV	NIEAS
Wuqi	TLD	TESV	NIEAS	TLD	TESV	RSS
Ganquan	TLD	TESV	NIEAS	TLD	NIEAS	AP
Fuxian	TLD	TESV	NIEAS	TLD	NIEAS	RSS
Luochuan	TLD	TESV	NIEAS	TLD	TESV	RSS
Yichuan	TLD	TESV	NIEAS	TLD	NIEAS	TESV
Huanglong	TLD	TESV	NIEAS	TLD	TESV	NIEAS
Huangling	TLD	TESV	RSS	TLD	TESV	NIEAS
Yuyang	TLD	RSS	NIEAS	RSS	TLD	AP
Hengshan	TLD	TESV	NIEAS	TLD	TESV	RSS
Shenmu	TLD	RSS	LRR	RSS	AP	GYPU
Fugu	TLD	TESV	RSS	RSS	NIEAS	GYPU
Jingbian	TLD	TESV	RSS	TLD	RSS	TESV
Dingbian	TLD	TESV	NIEAS	TLD	TESV	RSS
Suide	TLD	TESV	NIEAS	TLD	TESV	NIEAS
Mizhi	TLD	TESV	NIEAS	TLD	TESV	NIEAS
Jiaxian	TLD	TESV	NIEAS	TLD	TESV	AP
Wubao	TLD	TESV	NIEAS	TLD	TESV	NIEAS
Qingjian	TLD	TESV	NIEAS	TLD	TESV	RSS
Zizhou	TLD	TESV	NIEAS	TLD	TESV	NIEAS

Note: TESV represents total ecosystem service value; TLD represents the transportation land density; NIEAS represents the number of industrial enterprises above the scale; RSS represents the ratio of social security spending to GDP; LRR represents the land reclamation rate; AP represents the annual precipitation; GYPU represents the grain yield per unit area.

## Data Availability

The data presented in this study are available on request from the corresponding author. The data are not publicly available because part of them are being used in other studies that have not yet been publicly published.
